# Theca cells: active orchestrators of ovarian aging

**DOI:** 10.1186/s12958-026-01593-2

**Published:** 2026-07-20

**Authors:** Hongbo Wu, Zhao Zhang, Qianyi Huang, Bo Liu, Lidan Liu

**Affiliations:** 1 The First Affiliated Hospital of Guangxi Medical University, Nanning, China; 2 Qinzhou Maternity and Child Healthcare Hospital, Qinzhou, China

**Keywords:** Theca cells, Ovarian aging, Cellular senescence, Steroidogenesis, Premature ovarian insufficiency

## Abstract

Ovarian aging is characterized by the progressive depletion of the follicular reserve and deterioration of reproductive endocrine function, yet its cellular basis has been disproportionately attributed to oocyte and granulosa cell decline. Theca cells — the principal androgen-producing cell type of the ovarian follicle — have remained critically underappreciated in this context. Operating through the classical two-cell, two-gonadotropin model, theca cells supply androgen precursors that granulosa cells aromatize into estradiol, and their dysfunction therefore impairs estrogen biosynthesis irrespective of granulosa cell competence. During ovarian aging, theca cells undergo quantitative decline, transcriptional reprogramming, and progressive steroidogenic impairment. Single-cell transcriptomic analyses reveal age-dependent upregulation of senescence pathways — including CDKN1A, NF-κB, and SASP-associated factors — alongside downregulation of FOXP1, a transcription factor that normally suppresses cellular senescence through direct repression of CDKN1A. Senescent theca-interstitial cells elaborate pro-inflammatory mediators, most notably CCL5, TNF-α, IL-1β, and IL-6, which suppress steroidogenic gene expression, induce granulosa cell apoptosis, and promote ovarian fibrosis, collectively accelerating follicular atresia. At the molecular level, mitochondrial dysfunction driven by aberrant GSK3β activation impairs oxidative phosphorylation and steroidogenic capacity, while dysregulation of the PTEN/PI3K/Akt/FOXO1 axis uncouples theca cells from LH-dependent gonadotropin signaling. Theca cell dysfunction further underlies distinct clinical entities, including premature ovarian insufficiency, diminished ovarian reserve, and polycystic ovary syndrome. Emerging therapeutic strategies — encompassing mesenchymal stem cell-derived exosomes, senolytics, mitochondria-targeted interventions, and Wnt/β-catenin pathway modulation — have demonstrated efficacy in restoring theca cell function in preclinical models. This review repositions theca cells as active, bidirectionally regulated participants in ovarian aging—integral to, rather than upstream of, the oocyte–granulosa–theca unit—and proposes theca-specific biomarkers and targeted interventions as priorities for future translational research.

## Introduction

### The global health burden of ovarian aging

Ovarian aging is defined as the progressive depletion and functional deterioration of the primordial follicle pool. It represents the earliest organ-level aging event in the female body, with ovarian functional decline preceding that of other organ systems. Fertility diminishes markedly from the mid-thirties onward [1, 2]. The trajectory of follicular attrition is well characterized: from approximately 7 million germ cells at 20 weeks of gestation, the follicle pool contracts to approximately 1–2 million at birth, 300,000–400,000 at menarche, and fewer than 1,000 at the onset of menopause, which typically occurs at ages 51–52 years [2]. The rate of follicular depletion is not constant but accelerates with advancing age, with a particularly steep decline after the mid-thirties that substantially elevates the risks of aneuploidy and adverse reproductive outcomes [2]. According to the World Health Organization, infertility currently affects approximately 48 million couples worldwide, with ovarian aging accounting for a major proportion of female-factor infertility [2]. As global population aging intensifies and the mean age at first pregnancy continues to rise, the reproductive health consequences of ovarian aging have emerged as an escalating public health priority [2].

The consequences of ovarian aging extend far beyond reproductive failure. The hypoestrogenic state accompanying menopause precipitates a cascade of systemic sequelae, including osteoporosis, heightened cardiovascular risk, abdominal adiposity, diabetes, depression, and neurocognitive decline [1, 2]. The ovary has been described as the first organ to age, with its functional decline preceding broader systemic deterioration; later onset of menopause has been associated with lower mortality and prolonged longevity in observational studies [1, 3]. Although current therapeutic strategies — including antioxidant supplementation, dehydroepiandrosterone (DHEA), intraovarian platelet-rich plasma injection, and in vitro activation of primordial follicles — have demonstrated improvements in surrogate markers, these gains have not reliably translated into increases in live birth rates [4]. This translational gap reflects, in large part, an incomplete mechanistic understanding of all cellular constituents of the aging ovary.

### The central role of theca cells in follicular development

Follicular steroidogenesis operates through the well-established two-cell model, in which luteinizing hormone (LH) stimulates theca cells to convert cholesterol to androgens, which are subsequently transported across the basal lamina to granulosa cells and aromatized to estradiol under follicle-stimulating hormone (FSH)-induced CYP19A1 activity [5]. This strict division of labor means that theca cell dysfunction directly impairs estrogen biosynthesis, irrespective of granulosa cell competence. The functional contribution of theca cells extends beyond androgen substrate provision: they modulate granulosa cell steroidogenic capacity through paracrine androgenic signaling, while simultaneously receiving reciprocal regulatory input from paracrine factors — including oocyte-derived growth differentiation factor-9 (GDF-9) and bone morphogenetic protein 15 (BMP15), as well as granulosa cell-derived BMP4 and BMP6 — that drive theca cell differentiation and proliferation [5]. This obligate bidirectional interdependence ensures that age-related deterioration in either cell compartment propagates dysfunction to the other, amplifying the overall decline in follicular competence.

### The underappreciated role of theca cells in ovarian aging research

For decades, the scientific study of ovarian aging has been dominated by a granulosa cell- and oocyte-centric perspective — a focus that is not without justification, given that the age-related decline in oocyte meiotic fidelity, manifesting as aneuploidy rates of up to 60%, represents the most clinically consequential determinant of reproductive failure [3]. Nevertheless, this research emphasis has obscured the substantial contribution of the ovarian stromal compartment. Camaioni et al. (2022) [3] explicitly argued that ovarian aging is a complex and multifaceted process extending beyond changes in oocytes and granulosa cells. Stromal fibrosis, extracellular matrix (ECM) remodeling, and pro-inflammatory immune cell infiltration are recognized as early hallmarks of ovarian aging, potentially impairing follicle activation and oocyte quality through both mechanical and paracrine mechanisms.

Within this broader recognition of stromal contributions, theca cells occupy a paradoxical position: they are first recruited at the secondary follicle stage and become essential from that point onward, yet they remain among the least studied cell types in reproductive biology. As Vlieghe et al. (2023) [5] stated directly, early research “mainly focused on the interactions between granulosa cells and the oocyte, leaving theca cells unfairly forgotten in the understanding of ovarian physiology and pathogenesis.” This neglect carries concrete consequences: it has impeded the development of ex vivo follicle culture systems that faithfully recapitulate in vivo steroidogenesis, precluded the identification of theca cell-specific aging biomarkers, and left the androgen-deficient component of ovarian insufficiency without theca cell-specific targeted therapeutic strategies. Several factors have perpetuated this research gap: theca cells lack well-established surface markers, are routinely clustered within stromal cells in single-cell RNA sequencing datasets, and are systematically absent from primordial and primary follicles — the stages most amenable to experimental manipulation — thereby excluding them from many standard models of folliculogenesis [5].

This review synthesizes current knowledge on theca cell biology in the context of ovarian aging, encompassing molecular aging mechanisms, microenvironmental impact, associated pathological states, and emerging therapeutic strategies, with the aim of moving beyond the recurring observation that theca cells are underappreciated toward a mechanistically grounded synthesis: we integrate single-cell and spatial transcriptomic evidence, delineate the specific molecular axes (FOXP1–CDKN1A, PTEN/PI3K/Akt/FOXO1, GSK3β–mitochondrial dynamics, IL-6/PPARγ) through which theca cells participate in ovarian aging, and translate these into concrete, testable priorities—theca-specific biomarkers (e.g., INSL3) and targeted interventions—for the preservation of female reproductive longevity.

Beyond restating that theca cells are underappreciated, this review is distinguished from prior theca-focused reviews in three respects. First, it is organized specifically around ovarian aging rather than theca biology in general, reframing recent single-cell and spatial transcriptomic atlases of the aged human ovary through a theca-centric lens and connecting cell-type-resolved senescence signatures to defined steroidogenic and paracrine outputs. Second, it consolidates mechanistic findings that have emerged only recently and have not been integrated elsewhere—most notably the FOXP1–CDKN1A senescence axis, GSK3β-driven mitochondrial-dynamics failure, the IL-6/PPARγ theca-selective inflammatory pathway, and theca-targeted reparative mechanisms of mesenchymal stem cell exosomes—into a single causal framework linking theca dysfunction to follicular demise. Third, it translates this synthesis into an explicit, actionable agenda: a theca-specific biomarker (INSL3), candidate molecular targets (FOXP1, DLK1, GSK3β, PPARγ), and the experimental tools (theca-specific Cre drivers, targeted delivery systems) required to test them. The intended advance is therefore not advocacy for greater attention to theca cells, but a mechanistic and translational roadmap that the existing literature—predominantly granulosa- and oocyte-centric, or descriptive of theca biology outside the aging context—does not currently provide.

## Cell biology of theca cells

### Origin and differentiation

**Fig. 1:**
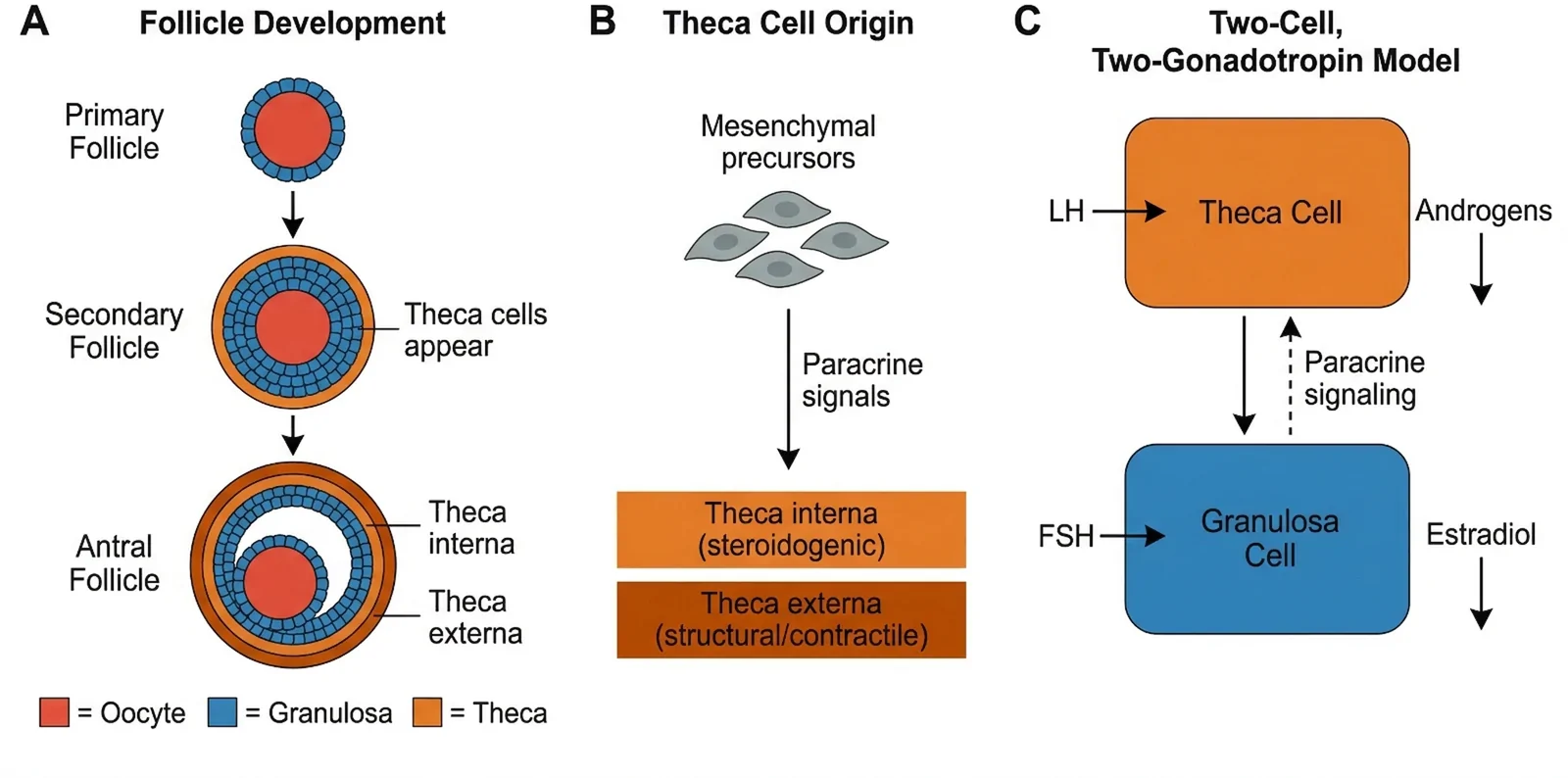
Theca cell biology and the two-cell, two-gonadotropin model. **A** Follicle development and theca cell differentiation: theca cells are first recruited at the primary-to-secondary follicle transition and form two layers—the theca interna and theca externa—around the granulosa cell compartment and oocyte as the follicle matures to the antral stage. **B** Theca cell origin and lineage specification: theca cells differentiate from ovarian stromal mesenchymal precursors under paracrine signals, giving rise to the steroidogenic theca interna and the structural/contractile theca externa. **C** The two-cell, two-gonadotropin model: luteinizing hormone (LH) drives androgen production in theca cells, and these androgens are transferred to granulosa cells and aromatized to estradiol under follicle-stimulating hormone (FSH) stimulation. The dashed bidirectional arrow denotes the reciprocal paracrine signaling between the two compartments, underscoring that theca and granulosa cells are mutually dependent rather than acting in a single direction. Colour key: red, oocyte; blue, granulosa cells; orange, theca cells. Abbreviations: LH, luteinizing hormone; FSH, follicle-stimulating hormone

Theca cells do not arise from a single progenitor pool; rather, they differentiate postnatally from mesenchymal precursors residing within the ovarian stroma, a lineage distinct from that of granulosa cells, which are thought to originate from the surface epithelium or, according to more recent lineage-tracing evidence, the rete ovarii. Theca cell recruitment is initiated at the transition from primary to secondary follicles, during which perifollicular stromal precursors are driven by paracrine signals — including stem cell factor, hedgehog ligands, and growth differentiation factors — to differentiate into committed theca progenitors, a process that continues throughout the reproductive lifespan. Acquisition of steroidogenic identity in theca cells is accompanied by expression of the androgen-biosynthetic enzymes CYP11A1, 3β-HSD, and CYP17A1, thereby enabling these cells to fulfill their role as androgen-producing units within the two-cell, two-gonadotropin model [5] (Fig. 1). The mature theca layer typically comprises two functionally distinct compartments: the theca interna, composed of epithelioid steroidogenic cells that express LHCGR, CYP11A1, CYP17A1, and 3β-HSD and produce androstenedione and testosterone in response to LH stimulation; and the theca externa, composed of smooth muscle-like cells expressing contractile proteins such as ACTA2, which provide structural support and contribute to follicle rupture at ovulation. Following follicular atresia, theca interna cells are relatively resistant to apoptosis; a substantial proportion persists, is incorporated into the ovarian stroma (Fig. 1), and undergoes hypertrophy to form secondary interstitial gland cells — a steroidogenic cell population that expands progressively throughout the reproductive years [6].

### Cellular heterogeneity

The theca cell compartment is not a homogeneous population; its intrinsic heterogeneity has been progressively revealed through lineage tracing and single-cell transcriptomics. In transgenic mice, Miyabayashi et al. (2015) [7], employing animals in which EGFP expression was driven by the fetal Leydig cell enhancer (FLE) of the SF-1 gene, found that only approximately 16.4% of SF-1-positive steroidogenic cells in the adult ovary were EGFP-positive, indicating that theca cells and interstitial gland cells encompass at least two developmentally distinct subtypes — by analogy with the fetal and adult Leydig cell populations of the testis, which show a similar dual-origin pattern. At the level of folliculogenesis, the transcriptional profile of theca cells shifts dynamically with follicle stage: steroidogenic enzyme expression is relatively low during the primary follicle stage; upon entry into the antral phase, LHCGR, STAR, CYP11A1, and CYP17A1 are upregulated, conferring full steroidogenic competence; and the preovulatory LH surge triggers transcriptional reprogramming toward a luteinized phenotype. In one single-cell transcriptomic analysis of the human ovary, Wu et al. (2024) [8] identified five theca-stromal cell subtypes: a steroidogenic subtype in the medulla (STAR⁺, CYB5A⁺, where CYB5A serves as a cofactor for CYP17A1 lyase activity); a perifollicular ECM-producing subtype (COL1A1⁺, COL3A1⁺); a perivascular myofibroblast subtype (ACTA2⁺, TAGLN⁺); a stress-responsive subtype in the outer cortex (FBLN1⁺, CXCL2⁺); and an inflammation-like subtype (CD74⁺, HLA-DRB1⁺) potentially involved in post-ovulatory repair. Interstitial gland cells, as a specialized theca-derived population, express CYP11A1 but not the leukocyte marker CD45; with advancing age, these cells accumulate lipid droplets and adopt a foamy morphology, sustaining steroidogenic function through lipophagy, as demonstrated in a chinchilla rabbit model [6].

### Key signaling pathways

PI3K/Akt/PTEN pathway. This pathway is central to theca cell steroidogenesis and is referenced throughout this review. LH binding to LHCGR activates PI3K, which converts PIP2 to PIP3, subsequently activating PDK1 and Akt; PTEN acts as the principal negative regulator by dephosphorylating PIP3 back to PIP2. Theca cell-specific Pten knockout mice (tPtenMT) exhibit hyperactivation of the PI3K/Akt axis, resulting in elevated serum testosterone, antral follicle accumulation, and early-onset infertility — a phenotype that closely recapitulates human PCOS. Mechanistically, Akt-mediated phosphorylation of FOXO1 relieves its transcriptional repression of the Lhcgr promoter, thereby establishing a positive feedback loop that amplifies androgen production [9].

Wnt/β-catenin pathway. Aberrant activation of Wnt/β-catenin signaling in theca cells impairs folliculogenesis through paracrine mechanisms: hyperactivated Wnt/β-catenin induces upregulation of DKK1 in cumulus cells and oocytes, which in turn suppresses oocyte Wnt/β-catenin signaling via the NPPC/cGMP/PDE3A/cAMP cascade. CYP17A1-Cre; Axin1^fl/fl mice, in which β-catenin is constitutively activated in a theca cell-specific manner, recapitulate the phenotypes of follicular arrest and impaired fertility. Apoptotic vesicles derived from mesenchymal stem cells (MSC-apoVs) carry RNF43, which promotes degradation of Frizzled receptors on the theca cell surface, thereby downregulating aberrantly activated Wnt/β-catenin signaling; systemic administration of MSC-apoVs to 15-month-old aged mice effectively ameliorated ovarian function and fertility capacity [10].

LH/LHCGR axis. LHCGR coordinates theca cell steroidogenesis through both the cAMP/PKA and PI3K/Akt pathways, which are not fully independent: PI3K/Akt-mediated phosphorylation of FOXO1 relieves its transcriptional repression of the Lhcgr promoter, thereby modulating LHCGR abundance and amplifying LH responsiveness [9]. Single-cell transcriptomic analysis has revealed that the transcription factor FOXP1 is progressively downregulated with age in theca-stromal cells; its capacity to suppress cellular senescence through direct binding to the CDKN1A (p21) promoter is consequently attenuated, leading to impaired ovarian cell proliferation and accelerated cellular senescence [8]. Secretion of the theca-specific peptide INSL3 is reduced in women with diminished ovarian reserve and correlates positively with established ovarian reserve markers including AMH and AFC, suggesting its potential as a complementary biomarker of theca cell functional decline in women with DOR, though its standalone diagnostic value requires further validation [11].

## Changes in theca cells during ovarian aging

### Morphological and histological changes

Ovarian aging is accompanied by profound histological remodeling, most prominently affecting theca cells and their derivative structure, the interstitial gland. Díaz-Hernández and colleagues conducted a systematic investigation of chinchilla rabbit ovaries from animals aged 1 to 34 months, revealing fundamental alterations in the distribution and function of theca cells across the aging process. While species-specific differences exist (Section "Knowledge gaps and challenges"), these findings provide valuable insights into potential mechanisms in human ovarian aging. In the reproductively active ovary, theca interna cells serve as the primary steroidogenic compartment of the follicle; the mitochondrial enzyme CYP11A1 catalyzes the conversion of cholesterol to pregnenolone, constituting the rate-limiting first step in androgen biosynthesis. With advancing age, CYP11A1 expression in the theca interna of antral follicles declined markedly, indicating a progressive attenuation of steroidogenic capacity in the aging ovary [12].

Concurrently, secondary interstitial glands underwent substantial expansion, ultimately becoming the most abundant tissue component of the aged ovary. The interstitial gland is formed through hypertrophy of theca cells derived from atretic follicles and accumulates continuously throughout reproductive life, given that approximately 99% of follicles undergo atresia rather than ovulation, with the theca cells of each atretic follicle incorporated into the interstitial compartment. In the aged ovary, the interstitial gland supplants follicles as the dominant structural element, accompanied by conspicuous remodeling of the ovarian cortex and surface epithelium [12].

At the ultrastructural level, interstitial gland cells in the mature ovary accumulate abundant lipid droplets, imparting a foamy appearance consistent with the morphological hallmarks of lipophagy—the autophagic degradation of lipid droplets. These cells retain CYP11A1 expression while lacking the pan-leukocyte marker CD45, confirming their identity as steroidogenic rather than macrophage-derived cells. Notably, these foam cells exhibit low p21 expression, sustained PCNA positivity, and absence of the DNA damage marker γH2AX, indicating that they have not entered canonical replicative senescence. Although the interstitial gland is the earliest ovarian compartment to accumulate lipofuscin—a recognized marker of cellular aging—lipofuscin levels plateau after reproductive maturity, DNA-damaged cells do not accumulate, and proliferative cell numbers actually increase at 36 months of age. Collectively, these findings indicate that interstitial gland cells employ lipophagy to sustain steroidogenic function and actively resist replicative senescence. However, it is important to note that the broader theca-interstitial cell population exhibits heterogeneity in aging phenotypes: while secondary interstitial gland cells derived from atretic follicles maintain proliferative capacity, other theca-interstitial cell subpopulations in aged ovaries display stress-induced senescence markers [6].

### Transcriptomic and epigenetic changes

Single-cell transcriptomic technologies have enabled unprecedented resolution in characterizing the molecular changes of theca cells during ovarian aging. Wu and colleagues [8] published a landmark study in Nature Aging, performing concurrent single-cell RNA sequencing (scRNA-seq) and spatial transcriptomics (ST-seq) on 15 human ovaries stratified by age (young: 18–28 years; middle-aged: 36–39 years; advanced: 47–49 years), profiling a total of 92,965 ovarian cells and identifying 8 major cell types. Serum AMH declined from 3.76 ng/mL in the young group to 1.35 ng/mL in the middle-aged group and fell below the detection threshold (< 0.06 ng/mL) in the advanced group, providing a direct quantitative reflection of age-dependent ovarian reserve decline.

Within the theca and stromal (T&S) cell population—hereafter referred to as theca-stromal cells or theca-interstitial cells (TICs) interchangeably, reflecting their shared mesenchymal origin and overlapping transcriptional profiles—UMAP analysis resolved five distinct subtypes: subtype 1 (STAR⁺, CYB5A⁺), representing classical steroidogenic theca cells predominantly localized to the ovarian medulla; subtype 2 (COL1A1⁺, COL3A1⁺), perifollicular cells providing structural support; subtype 3 (ACTA2⁺, TAGLN⁺), myofibroblast-like cells distributed in the perivascular niche; subtype 4 (FBLN1⁺, CXCL2⁺), cortical cells associated with stress responses; and subtype 5 (CD74⁺, HLA-DRB1⁺, CCL4⁺), inflammatory fibroblast-like cells potentially involved in post-ovulatory repair. The proportions of subtypes 2 and 5 declined with age, consistent with reduced follicular density and diminished ovulatory activity in the older ovary [8].

Differential gene expression analysis revealed that genes upregulated in the aging ovary were enriched in pathways including cellular senescence, FOXO, IL-17, NF-κB, NOD-like receptor, p53, and PI3K–Akt signaling, whereas downregulated genes were associated with extracellular matrix–receptor interaction, estrogen signaling, and oxidative phosphorylation. CDKN1A (p21) was upregulated across all ovarian cell types with aging, a finding corroborated by spatial transcriptomic data demonstrating its broad increase throughout the ovarian cortex and medulla. Within T&S cells, the senescence-associated secretory factors HMGA1 (*r* = 0.87), PIM1 (*r* = 0.77), and CAV1 (*r* = 0.69) were all significantly upregulated with age, and NF-κB signaling was markedly activated in T&S cells of the advanced-age ovary [8].

At the level of transcriptional regulation, single-cell regulatory network analysis (SCENIC) identified the transcription factor FOXP1 as progressively declining in both granulosa cells and T&S cells with age, a finding validated by Western blotting and immunohistochemistry. Knockdown of FOXP1 in the granulosa cell line COV434 and in primary T&S cells enhanced SA-β-galactosidase staining, upregulated senescence-associated proteins (CDKN1A, γH2AX, pNF-κB), and reduced proliferating cell numbers. ChIP–qPCR confirmed direct binding of FOXP1 to the CDKN1A promoter, and dual-luciferase reporter assays demonstrated that FOXP1 transcriptionally represses CDKN1A. In granulosa cell-specific FOXP1 knockout mice, follicle numbers were reduced, serum AMH and estradiol were decreased, and litter sizes were diminished, recapitulating features of premature ovarian insufficiency. These results establish FOXP1 as a critical anti-aging factor that maintains ovarian cell homeostasis through transcriptional repression of CDKN1A [8].

Zhang and colleagues [13] performed single-nucleus RNA sequencing combined with spatial transcriptomics on 12 human ovaries spanning 12–54 years of age, revealing a pervasive impairment of mitochondrial oxidative phosphorylation (OXPHOS) across multiple cell types in the aging ovary. The study further identified a novel endothelial cell subtype—CLDN5⁺ blood endothelial cells—which, in contrast to other cell types that lose their transcriptional identity with aging, exhibited enhanced antigen-presenting capacity and inflammatory activity. Spatial mapping revealed progressive accumulation of immunoglobulin-expressing cells (IGHG1⁺/IGKC⁺) at the ovarian periphery with advancing age. Of particular note, the DLK1:NOTCH3 signaling axis between theca cells and this endothelial subtype was amplified during aging and may contribute to ovarian dysfunction; DLK1 was also found to be upregulated in granulosa cells from patients with premature ovarian insufficiency [13].

Zhao and colleagues [14] reviewed single-cell studies of follicular development and ovarian aging, highlighting that scRNA-seq has uncovered cell type-specific mechanisms previously obscured by bulk tissue analyses and underscored the heterogeneity of theca and stromal cell responses to aging stimuli.

At the epigenetic level, Voros and colleagues [15] systematically reviewed epigenetic alterations in ovarian function. Global DNA hypomethylation is a consistent feature of ovarian aging; DNA methylation profiles in women with diminished ovarian reserve and polycystic ovary syndrome differ substantially from those of normal controls. Among non-coding RNAs, miR-27a-3p and miR-15a-5p have been implicated in granulosa cell dysfunction and poor ovarian response, and alterations in follicular fluid and granulosa cell miRNA profiles correlate with oocyte maturation, ovarian reserve, and embryo implantation potential. Histone modifications are equally important: histone acetylation at the CYP19A1 locus regulates aromatase expression in granulosa cells, and the balance between histone acetyltransferases and deacetylases governs gene transcription in ovarian cells throughout reproductive life [15].

### Functional changes

The functional consequences of theca cell aging manifest principally in declining steroidogenic capacity and alterations in specific biomarkers.

INSL3 (insulin-like peptide 3), secreted predominantly by theca interna cells of antral follicles and luteal cells, serves as a theca cell-specific marker of ovarian reserve. It participates in regulating androgen biosynthesis by theca cells and promotes oocyte maturation by inducing GDF9 expression in the oocyte. Ozcan and colleagues [11] conducted a prospective study of 75 women undergoing IVF—25 with diminished ovarian reserve (DOR), 24 with unexplained infertility (UI), and 26 controls with normal ovarian reserve—and found that both serum INSL3 (*p* = 0.001) and follicular fluid INSL3 (*p* < 0.001) were significantly lower in the DOR group than in the UI and control groups. Serum INSL3 correlated positively with AMH and antral follicle count (AFC) and negatively with FSH. Prior studies have further demonstrated that serum INSL3 declines progressively with advancing ovarian insufficiency and correlates closely with total antral follicle count in women with PCOS—a surrogate measure of total theca cell mass. Taken together, INSL3 provides a direct readout of theca cell function, and its sustained decline likely reflects the progressive reduction in theca cell mass that accompanies ovarian aging, though causality remains to be definitively established [11].

At the molecular level, Marti and colleagues [16] demonstrated that resveratrol downregulates CYP17 and CYP21 protein expression in human adrenocortical H295R cells through post-transcriptional mechanisms, and suppresses steroidogenesis in rat ovarian theca cells by inhibiting PKB/Akt signaling, establishing PKB/Akt as a central regulatory node of androgen biosynthesis in theca cells and providing mechanistic support for the therapeutic use of resveratrol in hyperandrogenic conditions such as PCOS [16].

Integrating the transcriptomic and functional evidence, theca cell aging emerges as a self-reinforcing cascade: age-dependent downregulation of CYP17A1 attenuates androgen biosynthesis in theca cells, depriving granulosa cells of the substrate required for normal estradiol synthesis; the progressive loss of FOXP1 removes a critical transcriptional brake on cellular senescence, accelerating the transition of theca cells toward a state characterized by reduced INSL3 secretion, impaired androgen output, and a senescence-associated secretory phenotype. The decline in circulating INSL3 reflects the progressive reduction in total theca cell mass consequent upon follicular depletion. Viewed in its entirety, theca cell aging does not represent the failure of a single gene or pathway, but rather a systemic collapse of the transcriptional regulatory networks, signaling architecture, and cellular structural integrity that collectively sustain steroidogenesis.

## Mechanisms of theca cell aging

### Cellular senescence and the SASP

Unlike granulosa cells, which are continuously renewed with each follicular wave, theca-interstitial cells (TICs) persist in the ovarian stroma and accumulate senescent characteristics over time. The theca-interstitial compartment comprises heterogeneous subpopulations with distinct aging trajectories: while secondary interstitial gland cells derived from atretic follicles resist replicative senescence (Section "Morphological and histological changes"), the broader TIC population exhibits stress-induced senescence characterized by elevated SA-β-galactosidase activity and SASP factor secretion. Histological analysis of aged mouse ovaries (9 months) demonstrates a marked increase in SA-β-galactosidase activity specifically in the theca-interstitial compartment compared to young controls (3 weeks), establishing TIC senescence as a defining feature of ovarian aging [17]. The interstitial gland cells derived from atretic follicles—originating from the hypertrophy of theca interna cells—represent a permanent and accumulative structure in the ovary that may contribute to its aging. These cells can serve as both pro- and anti-senescent sources, reflecting the dynamic complexity of this compartment [6].

The SASP of TICs is characterized by elevated CCL5, TNF-α, IL-1β, and IL-6 [17, 18]. CCL5 is the most prominently elevated factor: immunohistochemistry confirms its upregulation in the theca-interstitial compartment of aged ovaries, and elevated CCL5 is detectable in both follicular fluid and systemic circulation of aged mice [17]. In vitro, recombinant CCL5 (10–100 pg/mL) dose-dependently attenuates follicle growth, survival, and estradiol secretion, inducing granulosa cell apoptosis through concurrent NF-κB activation and PI3K/AKT suppression, with upregulation of BAX and downregulation of BCL-2. CCL5 further reduces oocyte-specific factors ZP3 and GDF9 and decreases the proportion of oocytes reaching metaphase II, demonstrating that TIC-derived SASP impairs the entire follicular unit [17].

In theca cells themselves, TNF-α, IL-1β, and IL-6 suppress steroidogenic gene expression (Lhcgr, Cyp17a1, Cyp11a1, Fshr, Cyp19a1), reducing androgen output and depriving granulosa cells of the substrate required for estrogen synthesis. All three cytokines upregulate Tgfb1, and IL-1β and IL-6 drive type III collagen deposition, contributing to ovarian fibrosis [18].

### Mitochondrial dysfunction and oxidative stress

Theca cells depend heavily on mitochondrial OXPHOS to sustain the energy demands of steroidogenesis, and this capacity is progressively compromised during aging. Aberrant GSK3β activation in aging theca cells disrupts the fusion–fission balance by enhancing DRP1 recruitment to mitochondria while inhibiting MFN1 and MFN2, producing fragmented mitochondria with reduced membrane potential, impaired respiratory capacity, and elevated ROS output [19]. Treatment with human umbilical cord mesenchymal stem cells (hUCMSCs) or pharmacological GSK3β inhibition restores mitochondrial dynamics, improves membrane potential, and rescues steroidogenic capacity in aged theca cells, identifying GSK3β as a tractable therapeutic target [19].

Complementarily, NR4A1 dysregulation in aging theca cells alters mitochondrial apoptotic signaling; hUCMSC treatment reduces theca cell apoptosis by stabilizing the NR4A1–mitochondrial pathway and preventing cytochrome c release [20]. The resulting decline in ETC complex activity and mitochondrial membrane potential directly impairs steroidogenic capacity in theca cells, reducing androgen substrate availability for granulosa cells [19].

### Inflammatory signaling

The IL-6/PPARγ axis represents the most theca cell-specific inflammatory pathway identified to date. Single-cell RNA sequencing reveals that IL-6Rα is highly and selectively expressed in theca-interstitial cells and nearly absent in granulosa cells, making TICs the primary ovarian target of circulating IL-6 [21]. IL-6 signaling in TICs suppresses PPARγ, reducing testosterone synthesis and downregulating HSD17B1, CYP17A1, and HSL in a dose-dependent manner. The functional consequences extend beyond TICs: conditioned medium from IL-6-treated theca cells impairs granulosa cell estradiol production, induces granulosa cell apoptosis, and restricts follicle growth in co-culture—effects fully reversed by the PPARγ agonist GW1929 [21].

In vivo, IL-6 neutralization in PM2.5-exposed mice restores serum AMH, healthy follicle counts, estradiol, and testosterone levels, and reverses downregulation of steroidogenic enzymes and PPARγ [21]. In a cohort of 215 women spanning normal ovarian reserve, DOR, and POI, serum IL-6 negatively correlates with AMH (*r* = − 0.4020, *P* < 0.0001) and testosterone, and positively with FSH (*r* = 0.4378, *P* < 0.0001), establishing IL-6 as a clinically relevant biomarker of theca cell dysfunction and ovarian aging [21].

**Fig. 2:**
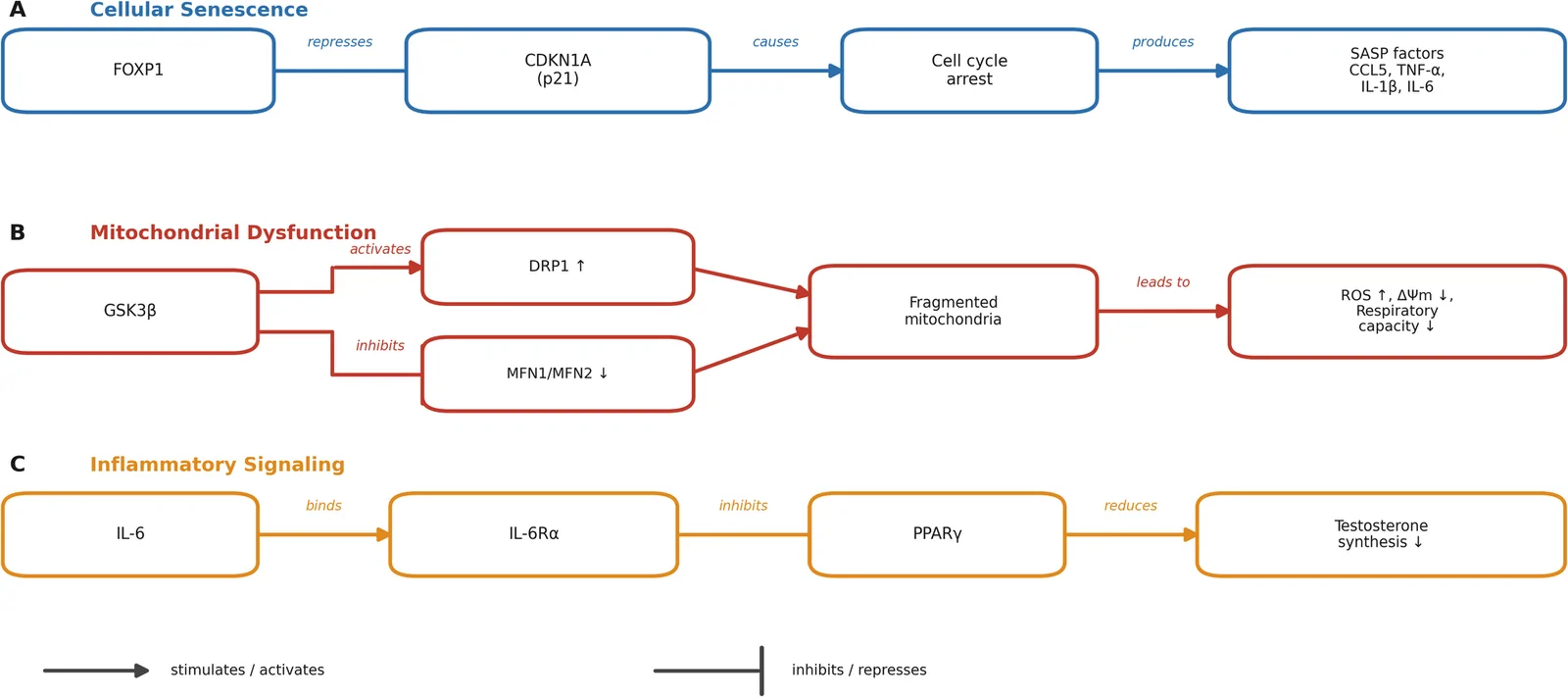
Mechanisms of theca cell aging. Three convergent pathways drive theca cell dysfunction during ovarian aging. Throughout, pointed arrows (→) denote stimulation or activation and bar-headed connectors (⊣) denote inhibition or repression. **A** Cellular senescence and the SASP: age-related loss of FOXP1 relieves its repression of CDKN1A (p21), driving cell-cycle arrest and acquisition of a senescence-associated secretory phenotype (CCL5, TNF-α, IL-1β, IL-6). **B** Mitochondrial dysfunction: aberrant GSK3β activation promotes DRP1-mediated fission while inhibiting MFN1/MFN2-mediated fusion, yielding fragmented mitochondria with increased ROS, reduced membrane potential (ΔΨm), and diminished respiratory capacity. **C** Inflammatory signaling: IL-6 binding to its receptor IL-6Rα on theca cells inhibits PPARγ, reducing testosterone synthesis. Abbreviations: SASP, senescence-associated secretory phenotype; ROS, reactive oxygen species; ΔΨm, mitochondrial membrane potential

The aging ovarian microenvironment is further shaped by tissue-resident macrophages exhibiting M2 activation status, which localize to the theca cell layer and corpus luteum and promote vascularization through angiogenic factor secretion to support steroidogenesis. Disruption of this immune–stromal crosstalk under inflammatory conditions contributes to follicular dysfunction [22] (Fig. 2).

### Genetic factors governing theca cell aging

A theca-centric interpretation of genetic contributions to ovarian aging was advanced by Rodgers and Laven (2020), who proposed the theca-layer hypothesis of premature ovarian insufficiency (POI) [23]. By integrating transcriptomic data from theca cells with POI-associated gene lists, they identified 38 cell-cycle and DNA-replication genes that are downregulated more than four-fold in the theca interna during follicular atresia. Twelve of these genes overlap with established POI loci, including BRCA1, FANCI, KNTC1, RAD51AP1, CDK1, CHEK1, and members of the MCM2–6 and POLE2 families [23]. Because most of these genes are also expressed in oocytes, granulosa cells, and tissues outside the ovary, this overlap does not by itself localize the pathogenic effect to theca cells; shared expression cannot be equated with theca-specific causation. The convergence is best interpreted as indicating that theca cell dysfunction may contribute to—rather than singularly drive—accelerated follicular exhaustion in genetically susceptible women, plausibly alongside concurrent oocyte and granulosa cell deficits. Establishing a theca-specific contribution will require genes whose expression is restricted to the theca lineage, or variants for which theca dysfunction has been experimentally or clinically confirmed.

At the molecular level, the PTEN/PI3K/Akt/FOXO1 axis in theca cells represents one of the best-characterized genetic pathways linking ovarian aging to reproductive failure. PTEN, a phosphatase that antagonizes PI3K signaling, is abundantly expressed in theca-interstitial (TI) cells throughout postnatal ovarian development [9]. Theca-specific deletion of Pten using the Cyp17iCre driver (tPtenMT mice) results in constitutive Akt activation, hyperphosphorylation of FOXO1 (at T24 and S256), and its cytoplasmic sequestration—relieving FOXO1-mediated repression of Lhcgr and thereby de-repressing LH receptor expression in TI cells [9].

The consequence is a paradox: despite elevated androgen biosynthesis capacity, tPtenMT females exhibit reduced ovulation by 2–3 months of age and progressive subfertility, with disrupted estrous cyclicity [9]. Mechanistically, FOXO1 occupies multiple regulatory regions of the Lhcgr promoter (confirmed by ChIP assay across eight putative FOXO1-binding sites, two of which showed direct occupancy), and its displacement by Akt-driven phosphorylation de-represses LH receptor expression, dysregulating the gonadotropin signaling required for normal folliculogenesis [9]. Pharmacological inhibition of Akt (API-2) in tPtenMT TI cells restores FOXO1 nuclear localization and rescues Lhcgr transcription, confirming the pathway’s causal role [9].

These findings position the PTEN→Akt→FOXO1→LHCGR axis as an important regulator of theca cell competence. The theca-layer hypothesis and the PTEN/FOXO1 mechanistic model together suggest that theca cell genetic integrity may be one contributor to—rather than the sole determinant of—reproductive aging tempo, acting in concert with established oocyte- and granulosa-intrinsic factors.

## Theca cell aging and follicular dysfunction

### Disruption of theca–granulosa cell communication

The two-cell model depends on seamless paracrine cooperation between theca and granulosa cells. Aging disrupts this cooperation through inflammatory signaling. In vitro studies demonstrate that pro-inflammatory cytokines characteristic of the aging ovarian milieu—TNF-α, IL-1β, and IL-6—suppress steroidogenic gene expression (Lhcgr, Cyp17a1, Cyp11a1, Fshr, Cyp19a1) in theca-interstitial cells (TICs), with each cytokine exerting a distinct inhibitory profile that impairs androgen production and downstream estradiol synthesis [18].

Beyond suppressing steroidogenesis, aging TICs actively secrete factors that damage neighboring cells. CCL5, a chemokine secreted by senescent TICs, directly inhibits preantral follicle development by activating NF-κB phosphorylation in granulosa cells while suppressing the PI3K/AKT survival pathway. This dual action tips the balance toward apoptosis and downregulates ZP3 and GDF9 expression, disrupting essential oocyte–granulosa communication [17].

The functional consequence is a cascade of follicular dysfunction. Reduced androgen delivery from dysfunctional theca cells limits estradiol synthesis even when granulosa cell aromatase activity remains intact. The resulting estrogen deficiency impairs granulosa cell proliferation and disrupts the androgen-dependent upregulation of FSH receptors that normally amplifies follicular growth. Thus, theca cell aging contributes to a self-reinforcing paracrine failure—steroidogenic impairment, androgen substrate insufficiency, estradiol deficiency, granulosa cell dysfunction, and follicular growth arrest—that operates bidirectionally rather than as a strictly linear, theca-initiated sequence, since theca specification and maintenance themselves depend on oocyte- and granulosa-derived signals.

### Theca cell-mediated fibrogenic remodeling and mechanical alterations

Theca cells are among the cell types that contribute to early ovarian fibrogenesis during aging, although their relative temporal primacy among stromal compartments remains to be established. Pro-inflammatory cytokines upregulate Tgfb1 expression and induce collagen III deposition specifically within the theca cell layer of preantral follicles, establishing a localized fibrogenic response that may seed broader age-related ovarian stromal fibrosis [18].

This fibrogenic remodeling has measurable mechanical consequences. Using three-dimensional quantitative micro-elastography (QME), Jaeschke et al. (2025) quantified the spatial elasticity patterns in murine ovaries across different age cohorts. Late-stage follicles exhibit a distinct ring of elevated elasticity in their outer region—coinciding with F-actin and α-SMA-rich theca cell layers—with an outer-to-inner elasticity ratio of approximately 2:1. This spatial pattern directly demonstrates the mechanical contribution of theca cells to follicular architecture [24].

Importantly, age-related mechanical changes are compartment-specific. Corpus luteum elasticity increases significantly with age, while follicle elasticity remains relatively stable. This suggests that post-ovulatory structures, which retain theca-derived cells, undergo more pronounced mechanical stiffening than developing follicles. Given that the theca layer is a primary site of fibrogenic signaling and that mechanical forces regulate follicular dormancy and activation through mechanotransduction pathways, age-related theca cell remodeling may progressively alter the mechanical microenvironment that governs follicular fate.

## Clinical implications

### Premature ovarian insufficiency (POI)

Theca cell dysfunction is increasingly recognized as a central pathological feature of premature ovarian insufficiency (POI). In autoimmune POI—which accounts for approximately 20% of cases—the theca interna of growing follicles is the primary site of lymphocytic infiltration, suggesting that theca cells are preferential targets of autoimmune-mediated ovarian destruction [25]. In chemotherapy-induced POI, three independent preclinical studies have delineated distinct but complementary mechanisms of theca cell injury. Chen et al. (2025) demonstrated that hUCMSC-derived exosomes target theca cells in cyclophosphamide (CTX)-treated POI rats and deliver miR-26a-5p, which targets PTEN to activate the PI3K/Akt/Nrf2/GPX4 axis, suppressing ferroptotic cell death and restoring steroidogenic function [26]. Luo et al. (2022) showed that cisplatin induces nuclear-to-cytoplasmic translocation of NR4A1 in theca cells, triggering the mitochondrial apoptosis cascade (Bax/cytochrome c/caspase-9/caspase-3); hUCMSC transplantation restored NR4A1 nuclear localization and recovered Cyp17a1 expression and testosterone synthesis [20]. Xiong et al. (2024) identified GSK3β overexpression in theca cells of CTX-POI mice as a driver of mitochondrial dynamics imbalance (impaired Mfn1/Mfn2 fusion, excessive Drp1 fission); hUCMSC transplantation normalized mitochondrial morphology and restored StAR and 17β-HSD expression [19]. Together, these studies establish ferroptosis, mitochondrial apoptosis, and mitochondrial dynamics failure as three targetable injury modes in POI theca cells.

### Diminished ovarian reserve (DOR)

Conventional ovarian reserve markers—AMH and AFC—reflect granulosa cell function and follicle quantity but provide limited information about theca cell integrity. Insulin-like peptide 3 (INSL3), produced exclusively by theca interna cells, offers a direct readout of theca cell mass and androgen biosynthetic capacity. Ozcan et al. (2025) demonstrated that both serum and follicular fluid INSL3 were significantly lower in DOR patients compared to controls (*p* < 0.001), with INSL3 correlating positively with AMH and AFC and negatively with basal FSH [11]. Notably, the proportional reduction in INSL3 was smaller than that of AMH, suggesting that theca cell mass declines more gradually than granulosa cell–derived AMH secretion. While INSL3 did not independently predict live birth rate in multivariate analysis, it may complement AMH in characterizing the theca–granulosa axis in women with DOR.

### Polycystic ovary syndrome (PCOS)

PCOS represents the opposite end of the theca cell dysfunction spectrum from POI: theca cell hyperfunction—rather than hypofunction—drives androgen excess, anovulation, and metabolic complications. Lan et al. (2017) provided direct genetic evidence by creating theca cell–selective Pten knockout mice, which developed elevated androgens, enlarged ovaries with antral follicle accumulation, and early fertility loss—a phenotype closely resembling human PCOS. The mechanism involves the PTEN/PI3K/Akt/FOXO1 axis described in Section "Key signaling pathways" and Section "Genetic factors governing theca cell aging" [9]. Fu et al. (2024) demonstrated that aberrant WNT/β-catenin activation specifically in theca cells disrupts folliculogenesis through the NPPC/cGMP/PDE3A/cAMP cascade; MSC-derived apoptotic vesicles (apoVs) deliver the WNT receptor inhibitor RNF43 to theca cells, normalizing WNT signaling and restoring follicle development in both PCOS and aging mouse models [10]. These findings position theca cell–specific PI3K/Akt and WNT/β-catenin modulation as mechanistically rational therapeutic strategies for PCOS.

### Early menopause and cardiovascular risk

A compelling hypothesis linking theca cell biology to the genetic architecture of menopause timing was advanced by Rodgers and Laven (2020). GWAS have identified 44 loci associated with age at natural menopause, of which 29 contain DNA damage response genes (BRCA1, FANCI, RAD51, CHEK2, MCM8, POLG). Transcriptomic profiling of bovine follicular atresia revealed that the theca interna downregulates 38 cell cycle and DNA replication genes by more than 4-fold during atresia, and 12 of these genes overlap with early menopause GWAS loci. This convergence supports the hypothesis that women carrying genome maintenance gene variants have theca cells more prone to DNA replication failure during atresia. This vulnerability leads to accelerated follicle loss and earlier menopause [23]. This model proposes theca cell vulnerability as one plausible contributor to early menopause—one that operates alongside, rather than upstream of, oocyte- and granulosa-intrinsic mechanisms—with potential implications for genetic counseling and fertility preservation.

## Therapeutic strategies

### Stem cell and extracellular vesicle therapies

Mesenchymal stem cells (MSCs) and their derivatives promote ovarian functional recovery primarily through paracrine signaling rather than direct differentiation [27, 28]. Three theca cell–specific reparative mechanisms have been established in POI models: (1) hUCMSC-derived exosomes target theca interstitial cells and deliver miR-26a-5p, which targets PTEN to activate the PI3K/Akt/Nrf2/GPX4 axis and suppress ferroptotic cell death [26]; (2) hUCMSC transplantation restores nuclear localization of NR4A1 in theca cells, thereby interrupting the Bax/cytochrome c/caspase-9/caspase-3 mitochondrial apoptosis cascade and recovering Cyp17a1 expression and testosterone biosynthesis [20]; (3) hUCMSC transplantation corrects GSK3β overexpression–driven mitochondrial dynamics imbalance in theca cells—characterized by impaired Mfn1/Mfn2-mediated fusion and excessive Drp1-dependent fission—restoring StAR and 17β-HSD expression [19]. In the extracellular vesicle domain, Tang et al. (2025) demonstrated that hUCMSC-Exos deliver let-7a-5p to specifically suppress excessive AMPK/mTOR-driven autophagy in theca-interstitial cells [29].

### Small-molecule and signaling pathway–targeted interventions

Multiple signaling axes with demonstrated regulatory roles in theca cell function offer tractable targets for small-molecule intervention. Along the NAD⁺/sirtuin axis, Matoba et al. (2024) showed in Leydig cells that SIRT3 deficiency elevates mitochondrial superoxide and impairs steroidogenesis, whereas SIRT3 overexpression confers protection; given that theca cells are the embryological counterpart of Leydig cells, SIRT3 activation may represent a targeted strategy for preserving theca cell steroidogenic capacity during aging [30]. Among antioxidant interventions, melatonin acts directly on theca cells via MT1/MT2 receptors, reducing intracellular ROS and enhancing StAR expression and progesterone secretion through the PI3K/Akt pathway [31].

### Anti-senescence and ovarian protection strategies

**Fig. 3:**
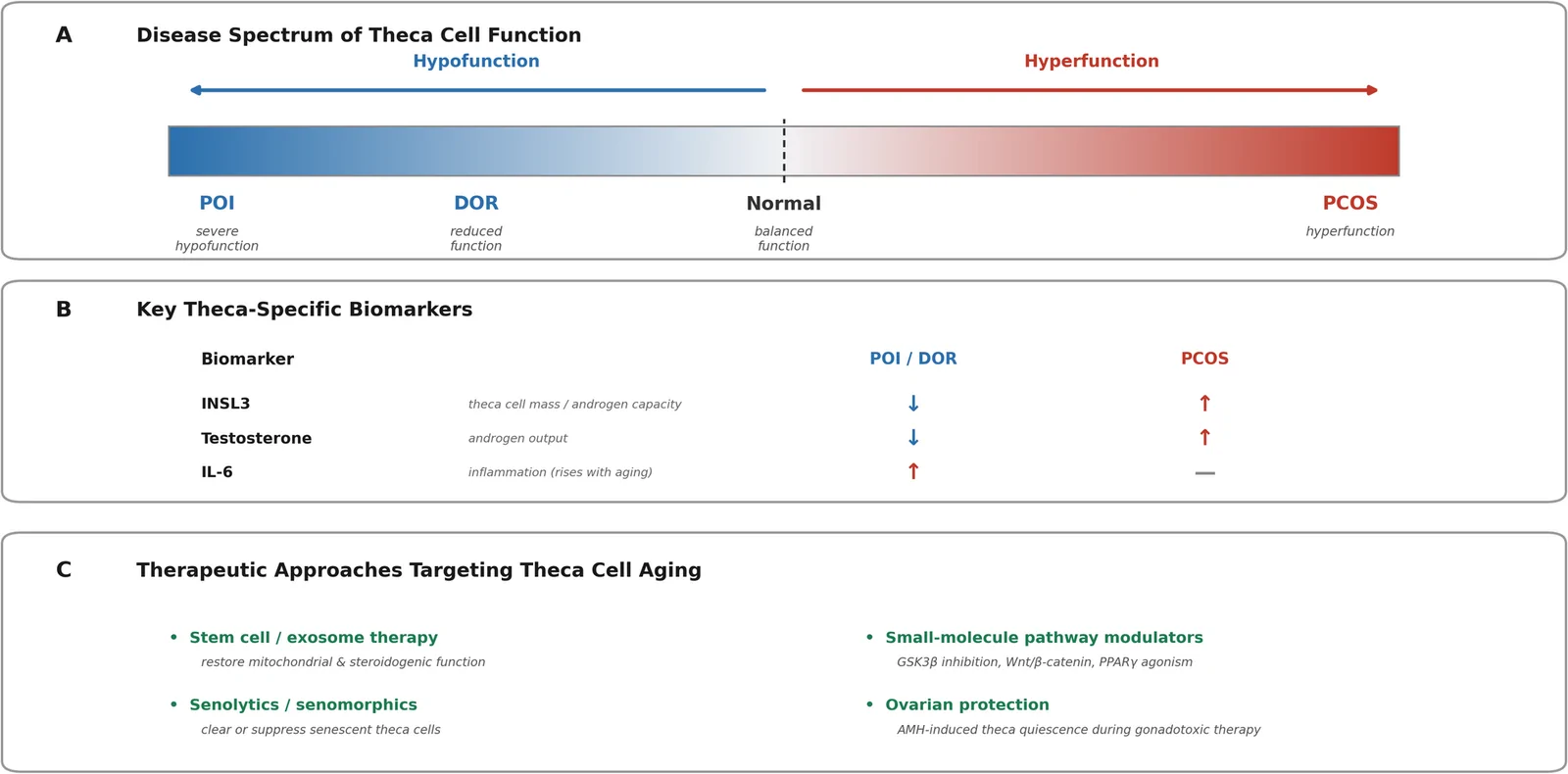
Clinical Implications of Theca Cell Dysfunction. **A** Theca cell dysfunction is represented not as a single linear scale but as a bidirectional spectrum centred on normal theca cell function: deviation toward hypofunction (reduced androgen output) underlies diminished ovarian reserve (DOR) and, more severely, premature ovarian insufficiency (POI), whereas deviation toward hyperfunction (androgen excess) characterizes polycystic ovary syndrome (PCOS). Both extremes are pathological, and “normal” denotes the balanced midpoint rather than an intermediate degree of dysfunction. **B** Theca cell-specific biomarkers and the direction of their change at each pole: INSL3 and testosterone decline in POI/DOR and rise in PCOS, while IL-6 rises with inflammatory/aging-associated theca dysfunction. **C** Therapeutic strategies targeting theca cell aging, with representative mechanisms. Abbreviations: POI, premature ovarian insufficiency; DOR, diminished ovarian reserve; PCOS, polycystic ovary syndrome

Cellular senescence in theca cells propagates SASP-mediated paracrine damage to the follicular microenvironment. Tang et al. (2025) comprehensively reviewed senescence across six ovarian cell types—including theca cells—and catalogued senotherapeutic strategies targeting the SASP, p16/p21 pathways, and telomere maintenance [32]. Senolytics eliminate existing senescent cells while senomorphics suppress the SASP of residual senescent cells; combinatorial approaches targeting both arms may offer superior efficacy for restoring theca cell function. Regarding ovarian protection during gonadotoxic therapy, AMH administration stalls theca progenitor differentiation and induces the DNA repair gene Id3, placing theca cells in a protective quiescent state; in a 6-month mating study, AMH-treated mice maintained significantly better fertility following doxorubicin exposure [33] (Fig. 3).

## Future directions and perspectives

### Technological advances

Single-cell and spatial multi-omics technologies are transforming our understanding of theca cell heterogeneity during ovarian aging. Wu et al. (2024) profiled human ovaries across reproductive stages using paired scRNA-seq and spatial transcriptomics, identifying five theca and stromal subtypes and revealing that FOXP1 decline drives CDKN1A-mediated senescence in these cells [8]. Zhang et al. (2026) applied spatial transcriptomics to characterize age-dependent changes in the ovarian microenvironment, uncovering spatially resolved gene expression patterns that distinguish aging theca cells from their younger counterparts [13]. Zhao et al. (2023) reviewed how single-cell sequencing has delineated distinct theca subpopulations with differential steroidogenic enzyme and inflammatory mediator expression in PCOS and POI contexts [14]. Looking forward, multi-omics integration combining scATAC-seq with spatial proteomics, three-dimensional ovarian organoid models, and AI-driven analytical frameworks will enable mechanistic dissection of theca cell aging at unprecedented resolution and provide tractable platforms for drug screening.

### Biomarker development

Current clinical markers of ovarian reserve (AMH, FSH, AFC) primarily reflect granulosa cell function and provide limited information about theca cell integrity. Insulin-like factor 3 (INSL3), produced almost exclusively by theca cells, has emerged as a promising theca-specific biomarker whose circulating levels decline with reproductive age and correlate with theca cell mass and androgenic capacity [11]. Clinical translation of theca-specific biomarkers will require standardized assay platforms, age-specific reference ranges, and prospective validation in multi-ethnic cohorts.

### Precision medicine

GWAS of age at natural menopause have identified numerous loci, with heritability estimates approaching 50%, implicating DNA damage response, apoptosis, and metabolic signaling pathways that are directly relevant to theca cell function [23]. Once companion diagnostics identify the dominant molecular lesion, targeted theca cell therapies such as FOXP1 gene delivery, DLK1 pathway modulation, or NAMPT activators could be deployed.

### Knowledge gaps and challenges

Several fundamental questions remain unresolved. First, a complete lineage map of theca cell origins—from progenitor specification through terminal differentiation—is lacking, hindering efforts to understand how developmental programming influences aging susceptibility. Second, the causal hierarchy among oxidative stress, mitochondrial dysfunction, NAD+ depletion, mTOR hyperactivation, and cellular senescence in theca cells has not been established; temporal dissection using inducible genetic models is needed. Third, the relationship between theca cell aging and systemic organ aging (cardiovascular, metabolic, neurological) remains poorly characterized despite epidemiological associations between early menopause and multi-organ disease risk. Fourth, translational gaps between rodent models and human biology are substantial, as mice lack a true menopause and differ in steroidogenic enzyme expression. Finally, the safety and long-term efficacy of proposed interventions—mTOR inhibitors, senolytics, and stem cell therapies—have not been established in reproductive-age women and require well-designed clinical trials with extended follow-up and theca-specific outcome measures.

Limitations of this review must also be acknowledged. First, the framing of theca cells as active participants in ovarian aging should not be read as implying unidirectional or upstream causation: theca cells are specified only after an oocyte–granulosa follicle has formed and depend continuously on oocyte- and granulosa-derived signals, so theca dysfunction is best understood as one interacting node within a reciprocally regulated follicular unit rather than as the initiating lesion of ovarian aging. Second, much of the genetic evidence linking theca cells to accelerated follicular loss rests on genes (e.g., DNA-damage-response and cell-cycle genes) that are also expressed in oocytes, granulosa cells, and extra-ovarian tissues; shared expression cannot localize causation to theca cells, and a theca-specific contribution will require genes with theca-restricted expression or variants for which theca dysfunction has been experimentally confirmed. Third, a substantial portion of the mechanistic data derives from rodent and other non-human models (e.g., mouse POI models, bovine atresia, chinchilla rabbit) that differ from humans in menopausal biology and steroidogenic-enzyme expression, so extrapolation to human ovarian aging remains provisional. Finally, human theca-cell data are still sparse—theca cells are frequently grouped with stroma in single-cell datasets and lack validated surface markers—so several of the conclusions synthesized here await confirmation with theca-resolved human studies. These limitations define, rather than undermine, the priorities outlined above.

## Conclusions

Theca cells are not passive bystanders but active, underappreciated participants in ovarian aging. Their role is reciprocal rather than unidirectional: although theca cells are specified only after an oocyte–granulosa follicle has formed and remain dependent on oocyte- and granulosa-derived signals, their dysfunction in turn propagates to these compartments. Their progressive functional decline impairs androgen substrate provision for estrogen biosynthesis, disrupts the growth factor milieu sustaining oocyte developmental competence, and propagates inflammatory SASP signals that accelerate the aging of neighboring granulosa cells and oocytes. This review has synthesized evidence demonstrating that theca cell aging operates through multiple interconnected mechanisms—oxidative stress and mitochondrial dysfunction, NAD+ metabolic decline, mTOR pathway dysregulation, cellular senescence, and epigenetic reprogramming—that converge to compromise both the steroidogenic and paracrine functions of these cells.

The therapeutic landscape has expanded considerably, with preclinical evidence supporting interventions ranging from mesenchymal stem cell-derived exosomes and NAD+ precursors to mTOR inhibitors, senolytics, and bioactive phytochemicals. Innovative delivery platforms—including lipid nanoparticles and GelMA hydrogel implants—are beginning to address the challenge of achieving therapeutic concentrations within the ovarian microenvironment. Meanwhile, single-cell and spatial transcriptomic atlases have provided unprecedented cellular and molecular maps of the aging ovary, revealing theca cell heterogeneity and intercellular communication networks that were invisible to bulk tissue analyses. The integration of these therapeutic modalities with precision medicine approaches—grounded in genetic risk stratification, theca-specific biomarkers such as INSL3, and multi-omics profiling—offers a realistic pathway toward individualized interventions that preserve ovarian function and extend the reproductive healthspan.

Future research should prioritize: (1) establishing the causal hierarchy among theca cell aging mechanisms through temporal genetic dissection; (2) developing theca cell-specific Cre driver lines and targeted delivery systems for mechanistic attribution; (3) validating theca-specific biomarker panels in prospective multi-ethnic cohorts; and (4) designing clinical trials with theca cell-relevant endpoints. Each priority is intended to advance understanding or intervention concretely: temporal genetic dissection would establish whether theca dysfunction precedes or follows oocyte/granulosa decline and thus whether it is an actionable point of intervention; theca-specific Cre drivers and delivery systems would permit causal attribution and cell-targeted therapy rather than whole-ovary exposure; biomarker validation (e.g., INSL3) would enable earlier detection of the androgen-deficient component of ovarian insufficiency that current granulosa-based markers (AMH, AFC) miss; and theca-relevant trial endpoints would test whether restoring theca function improves clinically meaningful outcomes rather than surrogate markers alone. As the global population ages and women increasingly defer childbearing, the imperative to develop safe, effective interventions for ovarian aging has never been greater. The field stands at an inflection point where molecular mechanisms are increasingly well-characterized, technological tools are maturing rapidly, and the first clinical trials are underway.

## Data Availability

No datasets were generated or analyzed during this study. All data discussed in this review are available in the cited published articles.
